# Antimicrobial resistance in shigellosis: A surveillance study among urban and rural children over 20 years in Bangladesh

**DOI:** 10.1371/journal.pone.0277574

**Published:** 2022-11-21

**Authors:** Sharika Nuzhat, Rina Das, Subhasish Das, Shoeb Bin Islam, Parag Palit, Md. Ahshanul Haque, Subhra Chakraborty, Soroar Hossain Khan, Dilruba Ahmed, Baharul Alam, Tahmeed Ahmed, Mohammod Jobayer Chisti, A. S. G. Faruque

**Affiliations:** 1 Nutrition and Clinical Services Division, International Centre for Diarrhoeal Disease Research, Bangladesh (icddr,b), Mohakhali, Dhaka, Bangladesh; 2 Laboratory Sciences and Services Division, International Centre for Diarrhoeal Disease Research, Bangladesh (icddr,b), Mohakhali, Dhaka, Bangladesh; 3 Department of International Health, Johns Hopkins Bloomberg School of Public Health, Baltimore, Maryland, United States of America; Regional Medical Research Centre Bhubaneswar, INDIA

## Abstract

Antimicrobial resistance against shigellosis is increasingly alarming. However, evidence-based knowledge gaps regarding the changing trends of shigellosis in Bangladesh exist due to the scarcity of longitudinal data on antimicrobial resistance. Our study evaluated the last 20 years antimicrobial resistance patterns against shigellosis among under-5 children in the urban and rural sites of Bangladesh. Data were extracted from the Diarrheal Disease Surveillance System (DDSS) of Dhaka Hospital (urban site) and Matlab Hospital (rural site) of the International Centre for Diarrheal Disease Research, Bangladesh (icddr,b) between January 2001 and December 2020. We studied culture-confirmed shigellosis cases from urban Dhaka Hospital (n = 883) and rural Matlab Hospital (n = 1263). Since 2001, a declining percentage of shigellosis in children observed in urban and rural sites. Moreover, higher isolation rates of *Shigella* were found in the rural site [1263/15684 (8.1%)] compared to the urban site [883/26804 (3.3%)] in the last 20 years. In both areas, *S*. *flexneri* was the predominant species. The upward trend of *S*. *sonnei* in both the study sites was statistically significant after adjusting for age and sex. WHO-recommended 1^st^ line antibiotic ciprofloxacin resistance gradually reached more than 70% in both the urban and rural site by 2020. In multiple logistic regression after adjusting for age and sex, ciprofloxacin, azithromycin, mecillinam, ceftriaxone, and multidrug resistance (resistance to any two of these four drugs) among under-5 children were found to be increasing significantly (p<0.01) in the last 20 years in both sites. The study results underscore the importance of therapeutic interventions for shigellosis by appropriate drugs based on their current antibiogram for under-5 children. These observations may help policymakers in formulating better case management strategies for shigellosis.

## Introduction

*Shigella* is the most common cause of invasive diarrhea. It remains accountable for increased morbidity and mortality rates in under-5 children in low- and middle-income countries [1]. The choice of an appropriate antibiotic for the treatment of shigellosis depends on the routine detection and monitoring of resistance patterns [2]. Clinical management, mostly of antimicrobial-resistant cases of shigellosis, is difficult without prior knowledge of the drug susceptibility patterns. Lack of such knowledge and inappropriate use of antimicrobials may lead to the onset of complications, requiring a prolonged hospital stay, and may ultimately result in fatal outcomes [3]. Globally, challenges for clinical management of shigellosis are emerging fast, more commonly in developing countries [4]. Multiple factors are involved in the emergence of antibiotic resistance, most often due to injudicious use of antibiotics resulting in resistant strains [5]. Drug resistance to *Shigella* infection develops by extrusion of drugs by active efflux pumps, decrease in cellular permeability, and over expression of drug-modifying and inactivating enzymes or target modification by mutation [6–8]. In addition to selective pressure and horizontal resistance-gene transmission, these different natures of antibiotic-resistance mechanisms contribute to an increase in the number of multidrug-resistant strains and cause conventional antibiotics to be highly incompetent against shigellosis within the shortest period [9]. Globally, it has been hypothesized that if necessary measure are not adopted to reduce the intensity of the rapidly expanding antimicrobial resistance, one person will die every three seconds by 2050 [10].

According to the WHO pocketbook, 1^st^ line of antibiotics for shigellosis in children is ciprofloxacin and 2^nd^ line of drugs is mecillinam /ceftriaxone [11, 12]. In this guideline, azithromycin was considered as 2^nd^ line drug for adults only. International Centre for Diarrhoeal Disease Research, Bangladesh (icddr,b) is monitoring the susceptibility pattern of *Shigella* isolates routinely as reported by the ongoing diarrheal disease surveillance system (DDSS) since its inception in treating shigellosis cases, seeking care from both urban (1979) and rural (1999) facilities. Alarming rates of resistance to ciprofloxacin were previously reported among the detected *Shigella* isolates [13], so azithromycin has currently been listed as a first [14] or second-line therapy [15] in some international guidelines for treating shigellosis in children. Consequently, cefixime has demonstrated effectiveness in treating shigellosis in both adult and pediatric patients [16, 17]. These different antibiotics are prescribed for the treatment of shigellosis, and enteric fever in children and it is an upsetting public health concern that resistance against this antimicrobial is also increasing over the period. Henceforth, we aimed to observe the changing pattern of resistance in the case of WHO-recommended 1^st^ and 2^nd^ line antibiotics used for shigellosis in children in Bangladesh.

## Materials and methods

### Ethical statement

For this study, data were extracted from the electronic database of hospital-based DDSS of both urban Dhaka Hospital and rural Matlab Hospital of icddr,b. DDSS is routine ongoing surveillance in hospitals of icddr,b located in Dhaka and Matlab, Bangladesh. The DDSS has the approval from the institutional review board of icddr,b (Research Review Committee and Ethical Review Committee) for data collection, analysis, and dissemination of the findings. At the time of enrolment into DDSS, verbal consent was obtained from the parents or the attending caregivers of each child, following hospital policy. Verbal consent was assured by showing the mark in the questionnaire to parents or caregivers. At the time of consenting, parents or caregivers were assured of ‘any risk being no more than minimal’, ‘their participation is voluntary’, ‘their rights to discontinue participation from the study’, and ‘the maintenance of strict confidentiality of disclosed information’. They were also informed about the use of anonymous data for analysis and using the results for improving patient management, conducting researches, and also for publication in leading peer-reviewed journals without disclosing the name or identity of their children.

### Study population and study site

Dhaka hospital and Matlab hospital of icddr,b provides care and free treatment to around 200,000 diarrheal disease patients each year and about 62% of them are children less than five years of age. In Dhaka hospital, the DDSS systematically (from every 50^th^ patient according to their hospital ID number) collected information, including age, sex, socio-demographic characteristics, clinical features, and common bacterial and viral etiology of diarrhea. For the Matlab hospital, patients from the Matlab HDSS (Health and Demographic Surveillance System) area were included in the study and stool samples were collected from each patient from this area attending in Matlab hospital. Parents or caregivers were interviewed by research assistants who collect demographic, socioeconomic, and clinical data. A physician documented the clinical findings including dehydration status. The stool sample was collected and submitted to the laboratory for microbiological evaluation. All relevant information was recorded into the electronic database as soon as possible. The present study’s analysis was limited to under-five children who were *Shigella* positive and enrolled in the DDSS from January 2001 to December 2020.

### Laboratory methods

A single, fresh stool specimen was collected from all enrolled patients and submitted immediately to the clinical microbiology laboratory in Dhaka/ Matlab. All stool samples were routinely screened for common enteric pathogens, including Enterotoxigenic *Escherichia coli*, *V*. *cholerae*, *Salmonella Shigella* spp., and rotavirus following standard laboratory procedures [18]. For *Shigella* identification, stools were inoculated onto MacConkey (MAC) and *Salmonella–Shigella* (SS) agar media plates. *Shigella* spp. were isolated and identified using standard laboratory methods [19] and grouped serologically by slide agglutination with specific antisera (Denka Seiken, Tokyo, Japan). Antimicrobial susceptibility of the isolated strains wasdetermined by the disc diffusion method as recommended by the Clinical Laboratory and Standard Institute, CLSI [formerly the National Committee for Clinical Laboratory Standards (NCCLS)] and already described in detail elsewhere [20]. The antibiotic discs used in this study (Oxoid, UK) included ciprofloxacin (5mg), mecillinam (25mg), Ceftriaxone (30mg). Before 2019 there was no information for Azithromycin in the CLSI guideline. For Zone size interpretive standards for disks, we used the WHO manual for the ‘Laboratory identification and Antimicrobial susceptibility testing of Bacterial Pathogens of Public health importance in the developing World’. For interpretation of test with 15 μg azithromycin disk, susceptibility zone ≥19 mm and resistance ≤15mm were considered (intermediate not noted). *E*. *coli* ATCC 25922 was used as the control for susceptibility studies [21].

### Definition

Multidrug resistance was defined by any of the two drugs out of ciprofloxacin, azithromycin, mecillinam, and ceftriaxone that were resistant to *Shigella* isolates.

### Data analysis

Data were analyzed using STATA version 15.0 IC (College Station, Texas). Statistical analyses included descriptive methods, including percentages of detection, species distribution, and resistance to ciprofloxacin, azithromycin, mecillinam, and ceftriaxone (WHO recommended 1^st^ and 2^nd^ line drug). Percentage of each species at each year were calculated from total number of *Shigella* positive isolates to assess the trend of each species with the 20 years period. For the strength of association (odds ratio) between *Shigella* positive isolates and change of the time (in year), multiple logistic regression was performed where each of the species was outcome variables and the time period in years was treated as exposure variable after adjusting for the age and sex.

The percentage of each species was documented in graphs separately for rural and urban sites. In the graphical presentation, 5 years prevalence of antimicrobial resistance to each drug was placed against every 5 years period for all *Shigella* species, *S*. *flexneri*, and *S*. *sonnei*. We did not include antimicrobial resistance of *S*. *boydii and S*. *dysenteriae*, as their percentage were low in both the sites. To assess the strength of association of resistance of antibiotics with time in years, adjusted odd-ratios (aORs) were calculated using multiple logistic regression where outcomes were ciprofloxacin resistance, azithromycin resistance, mecillinam resistance, ceftriaxone resistance, multidrug resistance (resistance to any of the two drugs) and independent variable was year, adjusted for age in months and sex of children.

## Results

**Fig 1:**
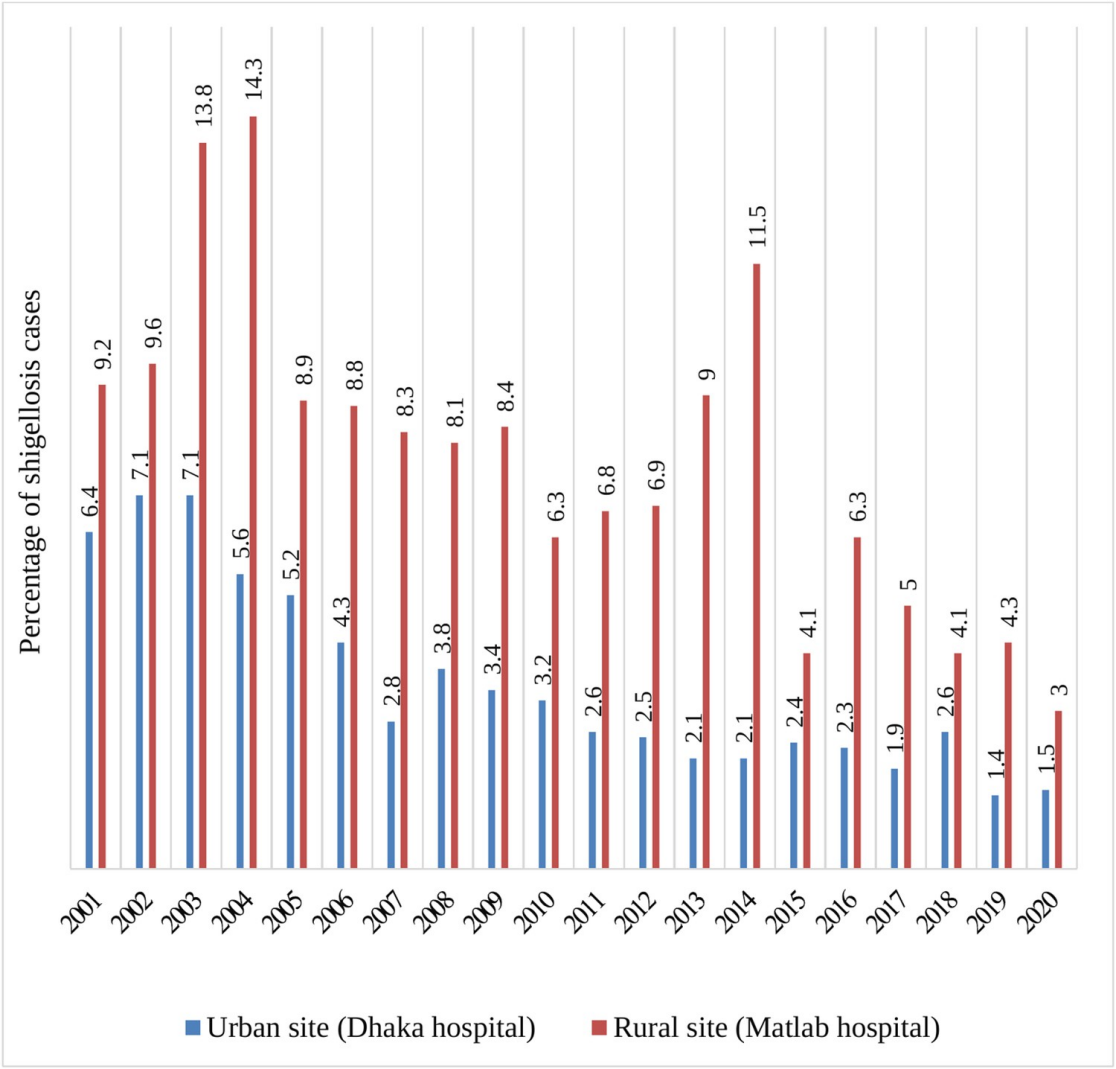
Percentage of shigellosis among under 5 children in last 20 years (2001–2020).

From 2001 to 2020, 26,804 and 15,684 under-5 children were included in Dhaka hospital and Matlab hospital surveillance systems, respectively. Of these, 883 and 1263 *Shigella* positive cases were enrolled in the urban and rural sites, respectively. Fig 1 shows the percentage of *Shigella* isolates in the last 20 years among under five children. Isolation rates of *Shigella* in the previous 20 years was 8.1% in the rural site and 3.3% in the urban site.

**Fig 2:**
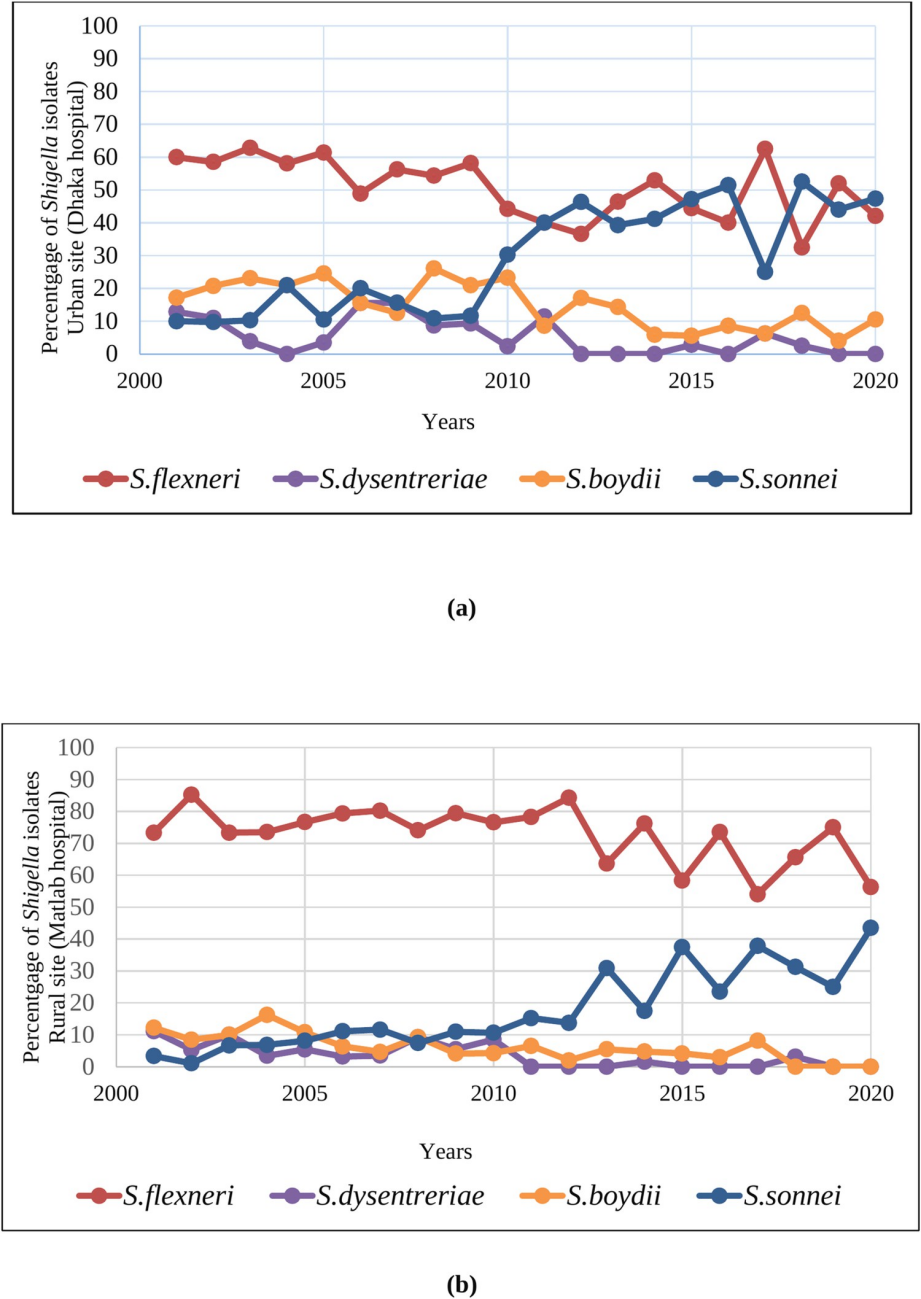
Percentage of different Shigella species in under 5 children in the urban site and rural site in last 20 years.

Fig 2(A) and 2(B) show the percentage of all species reported in the urban and rural sites in the last 20 years. In urban site *S*. *flexneri* was the predominant species in the early study period (up to 2010), whereas in the later periods (after 2010), detection of *S*. *sonnei* gradually increased. In rural site, *S*. *flexneri* was the predominant species during the entire study period, whereas in the later period, a gradual upward trend in the detection of *S*. *sonnei* was noted. But throughout the 20 years, there was a gradual decrease in the detection trend of *S*. *boydii* and *S*. *dysenteriae*.

In Table 1, multiple logistic regression shows a significant increase in the trend of detection of *S*. *sonnei* (aOR 1.13, 95% CI: 1.10–1.16; P <0.01) over 20 years period. Other *Shigella* species also show downward trend of isolation over the 20 years period after for adjusting age and sex.

**Table 1:** Trend of different *Shigella* species over the last 20 years in urban and rural sites.

	Urban site (Dhaka hospital)			Rural site (Matlab hospital)		
	Total isolates (n = 883) (%)	aOR (95% CI)	p-value	Total isolates (n = 1263) (%)	aOR (95% CI)	p-value
*S*. *flexneri*	463 (52.43)	0.96 (0.93–0.98)	<0.01	945 (74.82)	0.97 (0.94–0.99)	<0.01
*S*. *dysentreriae*	52 (5.89)	0.91 (0.86–0.96)	<0.01	60 (4.75)	0.87 (0.81–0.92)	<0.01
*S*. *boydii*	147 (16.65)	0.94 (0.91–0.97)	<0.01	96 (7.60)	0.91 (0.87–0.96)	<0.01
*S*. *sonnei*	221 (25.03)	1.13 (1.10–1.16)	<0.01	162 (12.83)	1.16 (1.12–1.20)	<0.01

aOR: adjusted odds ratio, adjusted for age and sex

Table 2 shows the percentage of isolates tested for all the recommended antibiotics for shigellosis in under-5 children. Throughout the 20 years, it was not possible to test all the antibiotics for all the *Shigella* isolates due to the availability of different antibiotic discs. It has been documented that 96.04% and 92.98% of samples were tested for ciprofloxacin and mecillinam respectively in the urban site. However, a lower number of samples were tested for azithromycin (38.96%) and ceftriaxone (40.32%). Similarly, from rural site, 95.01% and 94.14% samples were tested for ciprofloxacin and mecillinam, whereas around 20% samples were tested for azithromycin and ceftriaxone.

**Table 2:** Percentage of isolates tested for recommended antibiotics for shigellosis.

	Urban site (Dhaka Hospital)					Rural site (Matlab Hospital)				
	Total *Shigella* positive sample					Total *Shigella* positive sample				
	n (%)					n (%)				
	Percentage of samples tested for antibiotics (N = 883)					Percentage of samples tested for antibiotics (N = 1263)				
	Total *Shigella*	*S*. *flexneri*	*S*. *dysentreriae*	*S*. *boydii*	*S*. *sonnei*	Total *Shigella*	*S*. *flexneri*	*S*. *dysentreriae*	*S*. *boydii*	*S*. *sonnei*
**Ciprofloxacin**	848 (96.04)	452 (53.3)	52 (2.62)	146 (17.22)	198 (23.35)	1200 (95.01)	911 (75.92)	58 (4.83)	93 (7.75)	138 (11.50)
**Mecillinam**	821 (92.98)	433 (52.74)	47 (5.72)	132 (16.08)	209 (25.46)	1189 (94.14)	893 (75.11)	57 (4.79)	92 (7.74)	147 (12.36)
**Azithromycin**	344 (38.96)	155 (45.06)	9 (2.62)	38 (11.05)	142 (41.28)	259 (20.51)	174 (67.18)	1 (0.39)	8 (3.14)	76 (29.34)
**Ceftriaxone**	356 (40.32)	165 (46.35)	9 (2.53)	38 (10.67)	144 (40.45)	255 (20.19)	169 (66.27)	2 (0.78)	8 (7.74)	76 (29.80)

**Fig 3:**
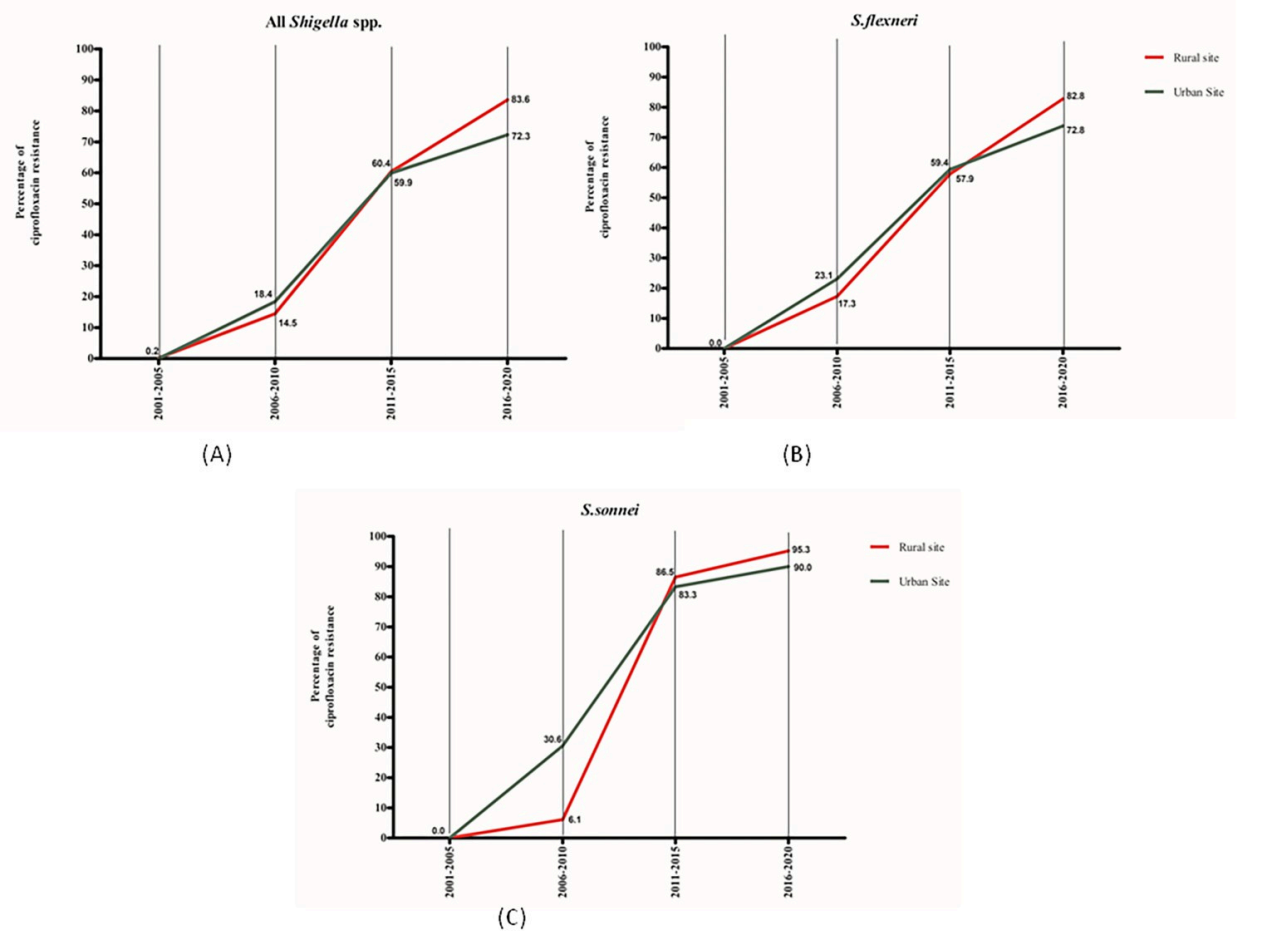
Percentage of ciprofloxacin resistance in shigellosis, 2001–2020.

The percentage of 1st line antibiotic ciprofloxacin resistance for shigellosis in under-5 children. It was observed that, although ciprofloxacin resistance started in the early decades, the resistance gradually increased to more than 70% in both the rural and urban site by 2020 (Fig 3).

**Fig 4:**
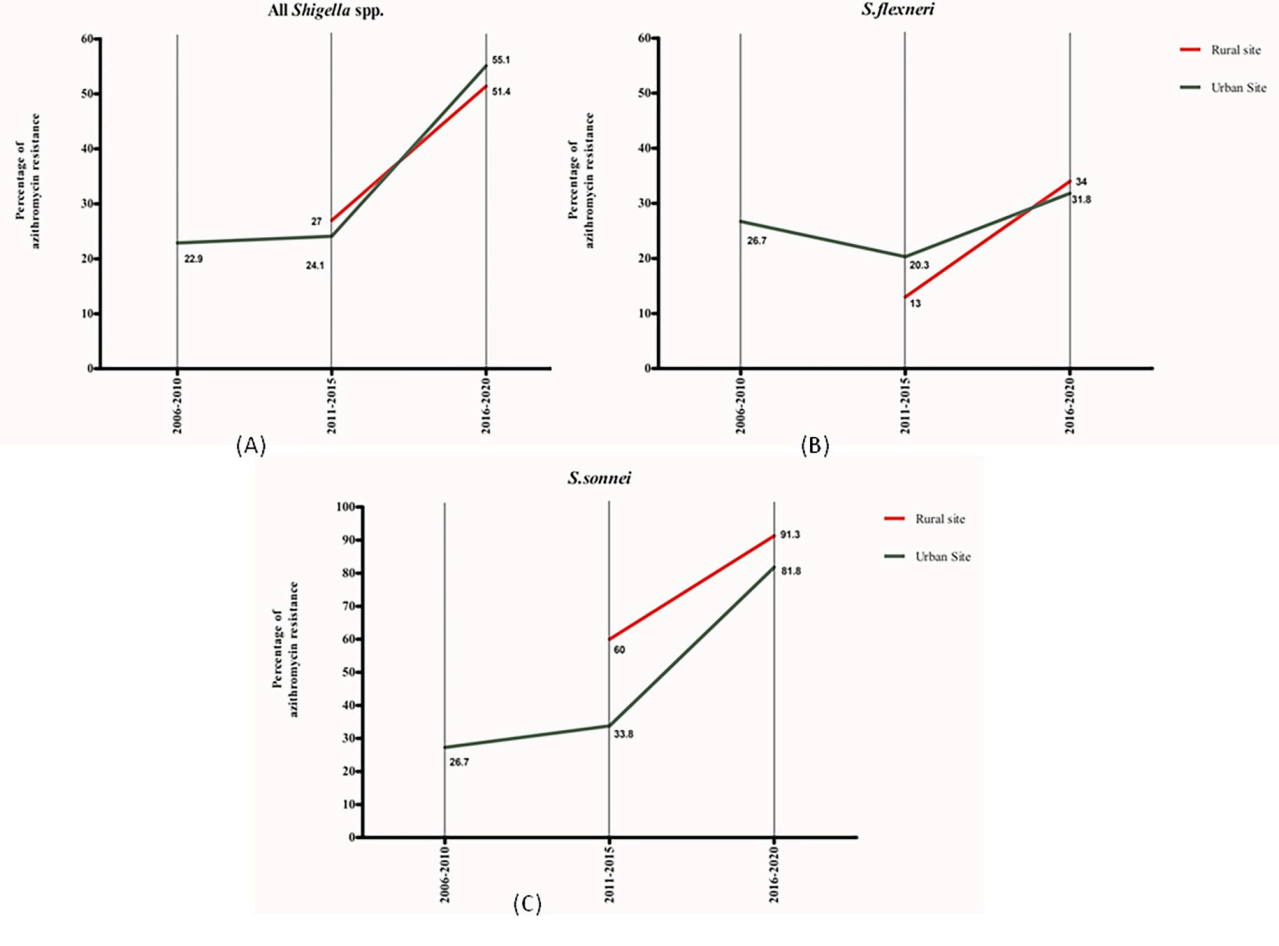
Percentage of azithromycin resistance in shigellosis, 2001–2020.

The percentage of resistance of azithromycin (2^nd^ line antibiotics) in under-5 children increased to more than 50%, both in the urban and rural sites. A comparatively higher percentage of resistance for azithromycin was observed in the case of *S*. *sonnei* compared to that of *S*. *flexneri* (Fig 4).

**Fig 5:**
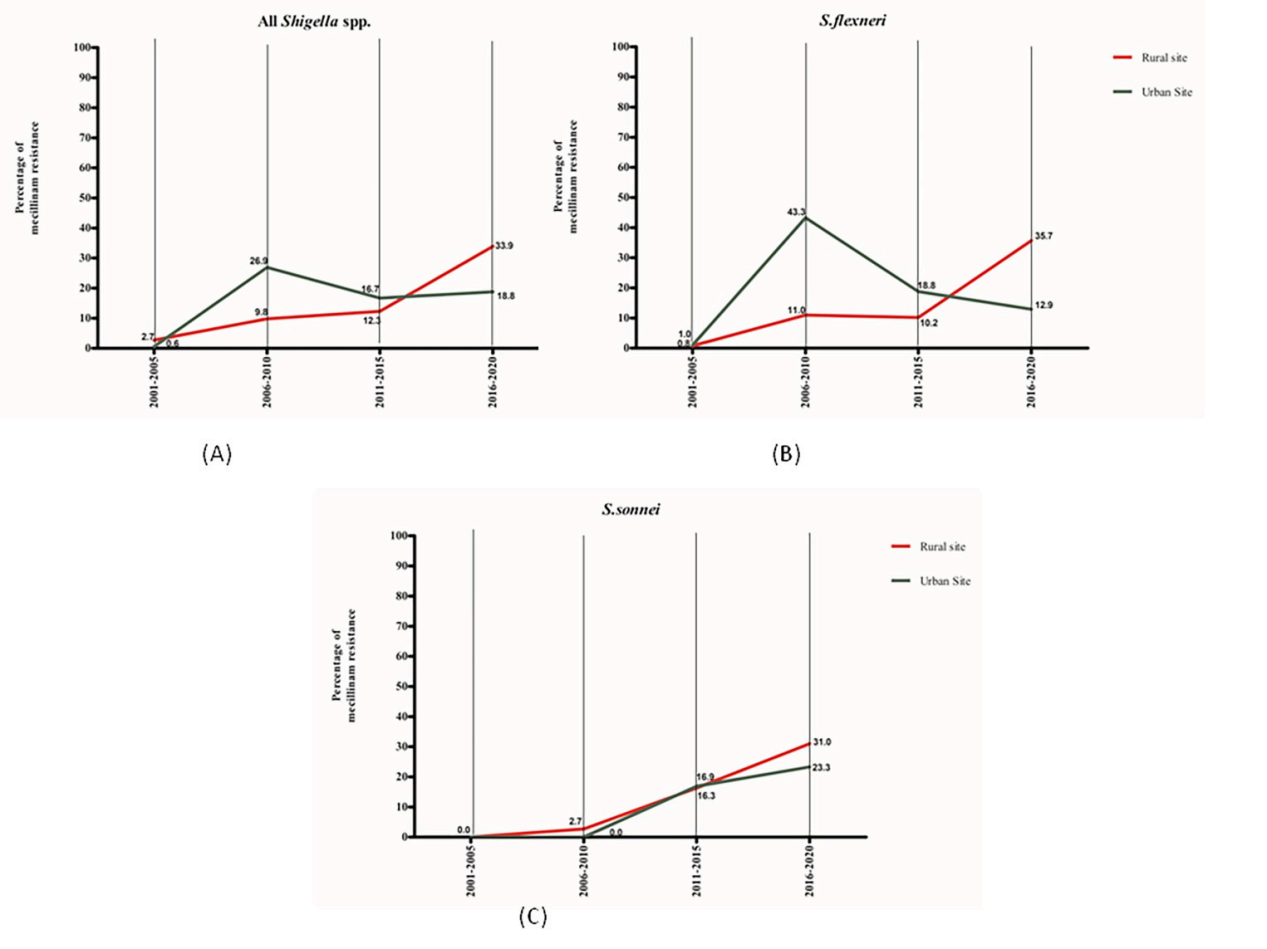
Percentage of mecillinam resistance in shigellosis, 2001–2020.

A diverse resistance pattern over the last 20 years was observed for mecillinam in the two study sites. Compared to the urban site, the rural site had a higher resistance in 2016–2020 (33.9% vs 18. 28%) against all *Shigella* isolates (Fig 5).

**Fig 6:**
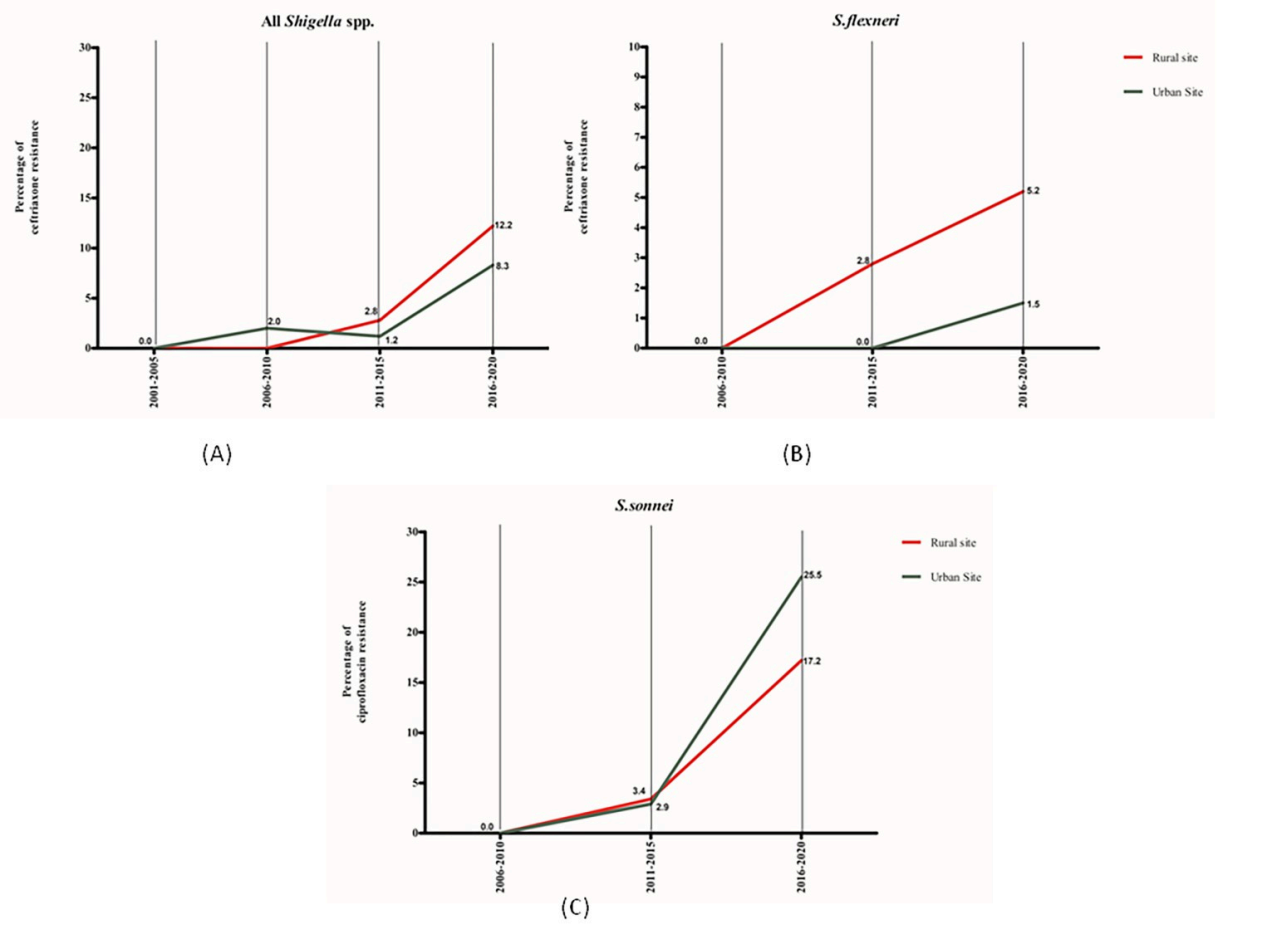
Percentage of ceftriaxone resistance in shigellosis, 2001–2020.

The ceftriaxone resistance started in the last decade (2011–2020) and gradually increased to more than 10% in the rural area, whereas in an urban area, it was 7.6% by 2020. Although only a few samples were tested for ceftriaxone, a comparatively higher percentage of resistance was observed for *S*. *sonnei* (Fig 6).

**Fig 7:**
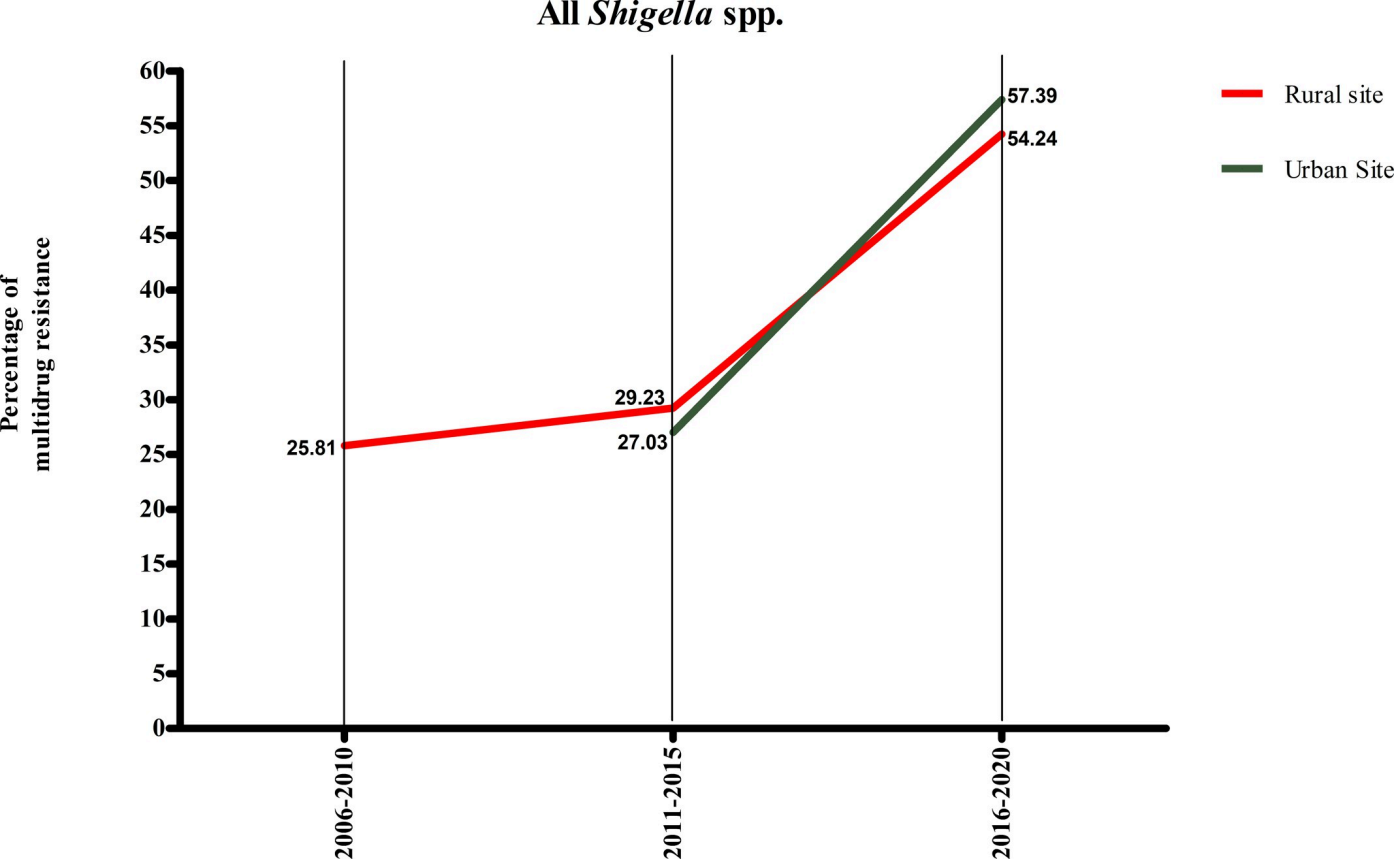
Percentage of multidrug resistance in shigellosis, 2001–2020.

After the early decade (2001–2010), a persistent rise in multidrug resistance in the case of shigellosis was observed in both the study sites (Fig 7). In the supplementary table (S1 Table), we have included the percentage of three and four drug resistance in urban and rural settings.

Tables 3 and 4 show the strength of association of antibiotic resistance in shigellosis among under-5 children in both urban and rural sites with the changes of time. After adjusting for age and sex, ciprofloxacin, azithromycin, mecillinam, ceftriaxone, and multidrug resistance increased significantly over time.

**Table 3:** Percentage of antibiotic resistance and association with year among under 5 children with shigellosis from 2001–2020 in urban site (Dhaka hospital).

Year	Ciprofloxacin resistance (%)	Azithromycin resistance (%)	Mecillinam resistance (%)	Ceftriaxone resistance (%)	Multidrug resistance (%)
2001	0.0	Test not done	1.49	Test not done	
2002	0.0	Test not done	0.0	Test not done	
2003	0.0	Test not done	1.35	Test not done	
2004	0.0	Test not done	0.0	Test not done	
2005	0.0	Test not done	0.0	Test not done	
2006	2.2	Test not done	0.0	0.0	
2007	6.2	Test not done	6.25	Test not done	
2008	27.7	Test not done	37.21	0.0	
2009	14.0	Test not done	48.78	0.0	
2010	47.50	22.86	37.50	2.63	25.81
2011	56.67	11.76	26.47	0.0	21.43
2012	45.45	25.0	9.76	0.0	21.88
2013	54.17	18.52	19.23	0.0	27.27
2014	64.52	27.59	13.33	3.33	43.48
2015	76.47	37.50	16.13	3.03	36.0
2016	80.0	57.14	14.71	3.13	61.29
2017	74.19	43.33	0.0	0.0	36.0
2018	65.71	56.0	32.26	13.51	52.0
2019	71.43	56.0	33.33	16.0	65.0
2020	68.4	66.67	11.11	10.53	58.82
aOR (95% CI)	1.41 (1.35–1.48)*	1.27 (1.17–1.37)*	1.13 (1.09–1.17)*	1.45 (1.16–1.81)*	1.21 (1.11–1.31)*

*p value <0.01

aORs were calculated using multiple logistic regression where outcomes were ciprofloxacin resistance, azithromycin resistance, mecillinam resistance, ceftriaxone resistance, multidrug resistance (resistance to any of the two drugs) and independent variable was year, adjusted for age in months and sex of children.

**Table 4:** Percentage of antibiotic resistance and association with year among under 5 children with shigellosis from 2001–2020 in rural site (Matlab hospital).

Year	Ciprofloxacin resistance (%)	Azithromycin resistance (%)	Mecillinam resistance (%)	Ceftriaxone resistance (%)	Multidrug resistance (%)
2001	0	Test not done	10.11	Test not done	
2002	0	Test not done	1.06	0/1	
2003	0.85	Test not done	0.85	Test not done	
2004	0	Test not done	0.88	Test not done	
2005	0.0	Test not done	1.39	Test not done	
2006	0.0	Test not done	6.35	Test not done	
2007	4.76	Test not done	6.98	Test not done	
2008	14.14	Test not done	9.80	Test not done	
2009	23.19	Test not done	16.42	Test not done	
2010	40.91	Test not done	6.52	Test not done	
2011	41.46	0.0	4.76	0.0	
2012	53.19	Test not done	8.89	Test not done	
2013	67.44	26.29	22	4.17	40.0
2014	68.42	23.33	11.54	3.39	20.93
2015	78.95	37.50	13.04	0.0	31.25
2016	76.47	55.88	36.36	2.94	60.61
2017	71.43	55.88	53.85	14.29	59.09
2018	92.86	50.0	44.44	16.1	73.91
2019	93.55	0.63	6.9	12.5	39.29
2020	91.67	56.25	25.0	18.75	55.56
aOR (95% CI)	1.59	1.21	1.91	1.40	1.21
	(1.51–1.68)*	(1.08–1.36)*	(1.15–1.24)*	(1.11–1.77)*	(1.05–1.40)*

*p value <0.01

aORs were calculated using multiple logistic regression where outcomes were ciprofloxacin resistance, azithromycin resistance, mecillinam resistance, ceftriaxone resistance, multidrug resistance (resistance to any of the two drugs) and independent variable was year, adjusted for age in months and sex of children.

## Discussion

Our observation from the DDSS on the detection of *Shigella* isolates among under-5 children warrants greater clinical vigilance regarding the use of appropriate therapeutic strategy, owing to the gradual antimicrobial resistance among the *Shigella* isolates. The most important observations involve a twofold higher detection rate of *Shigella* cases in rural site compared to urban site and an alarming rise of antimicrobial resistance in both sites over the last 20 years.

Different studies, including the Global Enteric Multicenter Study (GEMS) reported that in the rural study site, poor hygiene practices, not covering the water container, not use of handwashing substances, and children from low-income families are vulnerable to childhood shigellosis [22, 23]. Tube-well is the primary drinking water source for Bangladesh’s rural population [24]. A shallow tube well is likely to be contaminated with adjacent polluted water sources due to its shallowness [24]. Moreover, deep tube-well water users are susceptible to *S*. *sonnei* infection due to decreased water contamination with *Plesiomonas Shigelloides*, which shares similar antigenic characteristics to *S*. *sonnei* [23]. Probably these are the factors associated with a higher percentage of isolation of *Shigella* cases in the rural site.

In our study, *S*. *flexneri* was the predominant species in both urban and rural sites among all *Shigella* cases. However, *S*. *sonnei* demonstrated an increasing trend that was statistically significant. We did not detect any *S*. *dysenteriae* isolates in 2019–2020. Increasing detection of *S*. *sonnei* has also been reported in many countries, including Bangladesh [23]. It is observed from different studies that developed nations have a predominance of *S*. *sonnei* [25–27] whereas in recent years, an increased trend of *S*. *sonnei* has been observed in developing countries [28]. *P*. *shigelloides* is a gram-negative bacterium surviving in surface water that shares antigens with *S*. *sonnei* [29], and exposure to *P*. *shigelloides*, through contaminated drinking water, may immunize people to *S*. *sonnei* in developing countries despite exposure to contaminated drinking water [30]. The upward trend of *S*. *sonnei* infections can be explained by the reduced exposure of individuals from developing countries, including Bangladesh, to *Plesiomonas Shigelloides* (*P*. *shigelloides*) from drinking water in recent years [31]. The gross economic improvement in Bangladesh may be linked to an increase in the use of relatively safe deep tube-well water (DTW) and bottled water [32], and thereby the present study observed an enhancing trend of higher *S*. *sonnei* cases. Climate and environmental changes over time, as well as changes in innate characteristics of different serogroups and serotypes of Shigella, are additional explanation for this change [33].

Our study, covering the years 2001–2020, demonstrated the increasing antimicrobial resistance of *Shigella* isolates among under-5 children in both urban and rural diarrheal disease hospitals in Bangladesh. The resistance to antimicrobial agents that are commonly used to treat shigellosis in young children, namely: mecillinam, azithromycin, ciprofloxacin, ceftriaxone as well as multidrug resistance reached 33.9% vs. 18.8%, 55.1% vs. 51.4%, 83.6% vs. 72.3%, 12.2% vs. 8.3% and 47.7% vs. 43.1% in the rural and urban setups, respectively in last 5-year period. Previous studies have shown that the resistance rate observed in a hospital-based microbiology laboratory was similar to all *Shigella*-positive cases compared to community-based microbiology laboratories [2, 34]. This was expected almost all cases were community-acquired as the cultures were obtained on the admission samples. This suggests that the resistance rates found in the present study are typical of the scenario in the community.

Antimicrobial treatment is suggested for shigellosis to prevent further complications, shorten the duration of fever, reduce diarrheal output, and limit post-symptomatic fecal shedding, particularly in malnourished children living in low- and middle-income countries [35]. Unfortunately, resistance to antimicrobials appears to arise rather effortlessly in the case of *Shigella*. It may result from an unrestricted barrier for horizontal gene transfer between *Shigella* in urban and rural settings.

The current recommended first-line treatment for shigellosis is fluoroquinolones, such as ciprofloxacin, although, these also rapidly became the mainstay prescription for acute diarrhea in endemic regions [36]. The trend of resistance to ciprofloxacin is rising, probably due to its over-the-counter availability and common use. Asia is a reservoir for the rise and spread of resistant bacteria [37]. Specifically, ciprofloxacin-resistance among isolates of *S*. *sonnei* has risen as a single clone, especially in South Asia, before applying globally to Southeast Asia and Europe [38]. Such resistance depends on the gradual accretion of the triple mutations in chromosomal gyrA and parC. Moreover, horizontally transferred elements could help to establish emerging resistant clones [39].

A study conducted in Bangladesh reported that the rate of mecillinam resistance was significantly lower in Bangladesh [16]. We have also observed similar findings that showed comparatively lower resistance than other 2^nd^ line antibiotics for shigellosis, although yearly resistance to mecillinam showed fluctuation. The absence of pediatric formulation for mecillinam and poor compliance to recommended multiple doses with a bitter taste may have made mecillinam less susceptible to drug overuse, mostly in children. In addition, fluctuation in this drug’s steady supply might contribute to the change of resistance pattern.

Azithromycin has been considered to be the last oral choice for the treatment of bacillary dysentery. Decreased susceptibility to azithromycin was reported in recent years worldwide. Analyzing our antibiotic resistance data, we observed that azithromycin resistance increased almost four times from 2011 to 2020. A study from public health surveillance in Australia showed resistance to azithromycin is mediated by the plasmid pKSR100, which was recently revealed to be acquired in separate *S*. *sonnei* and *S*. *flexneri* 2a isolates [40]. Probably in Bangladesh, the selective pressure from azithromycin administration due to diarrhea-related symptoms might have contributed to the emergence of resistance in this organism.

Among hospitalized children with shigellosis, parenteral ceftriaxone is effective and usually recommended. *Shigella* was highly susceptible to ceftriaxone (>98%), which was introduced during the early decade (2001–2010) of the study period as a result of increasing resistance to mecillinam and ciprofloxacin [41]. In our study, among all *Shigella* cases, resistance to ceftriaxone increased over the period in both the study sites. However, we could not draw any conclusive remark on it because of the low number of samples tested. Very recently published research on shigellosis also observed increasing resistance to ceftriaxone [42, 43].

Multidrug resistance of shigellosis is commonly reported all over the world. The Centre for Disease Control (CDC) has categorized *Shigella* infections as a serious threat because of the increasing trend of drug resistance [44]. Resistance to ciprofloxacin, β-lactam antibiotics, or plasmid-mediated azithromycin resistance is of high concern [45, 46]. Our study observed that over the 20 years, multidrug resistance shigellosis has been increasing in both urban and rural settings, and the rate of this increase is more significant in the urban site.

In the last five years, there was a sharp rise of mono-drug and multidrug-resistant shigellosis in the rural site. Antibiotic resistance in urban and rural Bangladesh exhibited a trend of rising and falling in a simultaneous and synchronized manner [41]. A very recent article published on using data collected from 2017–2019 reported that the perception of a rural community on the use of antibiotics and AMR and it also showed the care-seeking changes in community-level [47]. A survey report from Bangladesh demonstrated irrational use of antibiotic among rural community [48]. Probably these explains the changes of antibiotic resistance pattern of rural site in the recent five years.

Our study has a few limitations warranting a careful interpretation of the results. We were able to evaluate only a limited number of antimicrobial susceptibility patterns of shigellosis due to the lack of availability of laboratory data which were extracted from the electronic database of hospital-based DDSS of Dhaka hospital and Matlab hospital. Moreover, the study was conducted in a diarrheal disease hospital setting in Bangladesh, so the results may not be generalizable for the more extensive and diverse population. Moreover, the issue of sub-clinical infections by *Shigella*, as reported in studies conducted in similar settings [49], was not considered for this study. To revise treatment guideline, antimicrobial susceptibility needs to be evaluated from community level.

In conclusion, our study findings identified changing pattern of *Shigella* species, and emerging resistance in *Shigella* isolates to WHO recommended 1^st^ and 2^nd^ line antimicrobial agents in the last 20 years in Bangladesh. Multidrug-resistant shigellosis is also gradually increasing both in urban and rural settings. physicians should be aware of the high rates of antimicrobial resistance to *Shigella* spp. in Bangladesh. Undoubtedly, the treatment of shigellosis among under-5 children demands careful and judicial use of antimicrobials to avoid rapid emergence and spread of resistance. At the same time, our study results underscore the need for continued monitoring and evidence-based alternative antibiotic regimens for multidrug-resistant *Shigella* infections.

## Supporting information

S1 Table Multiple antibiotic resistance (WHO recommended antibiotics) among under 5 children with shigellosis from 2001–2020. (DOCX)
