# Supplementary material for: Antimicrobial resistance in shigellosis: A surveillance study among urban and rural children over 20 years in Bangladesh
Source: PLoS One. 2022 Nov 21;17(11):e0277574. doi: 10.1371/journal.pone.0277574 (PMC9678309; doi:10.1371/journal.pone.0277574)
Supplement: S1 Table — (DOCX) [file pone.0277574.s001.docx]

Supplementary Table S1: Multiple Antibiotic resistance (WHO recommended antibiotics) among under 5 children with shigellosis from 2001-2020

| years | Any two antibiotics resistant  (any of the two drugs out of ciprofloxacin, azithromycin, mecillinam, and ceftriaxone)  n (%) | | Any three antibiotics resistant  (any of the three drugs out of ciprofloxacin, azithromycin, mecillinam, and ceftriaxone)  n (%) | | Any four antibiotics resistant  (any of the four drugs out of ciprofloxacin, azithromycin, mecillinam, and ceftriaxone)  n (%) | |
| --- | --- | --- | --- | --- | --- | --- |
|  | Urban Site (Dhaka hospital) | Rural Site (Matlab hospital) | Urban Site (Dhaka hospital) | Rural Site (Matlab hospital) | Urban Site (Dhaka hospital) | Rural Site (Matlab hospital) |
| 2010 | 5/31 (16.1) |  | 3/31 (9.7) |  | 0/31 |  |
| 2011 | 5/28 (17.9) |  | 1/28 (3.6) |  | 0 |  |
| 2012 | 6/32 (18.8) |  | 1/32 (3.1) |  | 0 |  |
| 2013 | 4/22 (18.2) | 5/15 (33.33) | 2/22 (9.1) | 1/15 (6.67) | 0 | 0/15 |
| 2014 | 9/23 (39.1) | 6/43 (13.95) | 1/23 (4.3) | 3/43 (6.98) | 0 | 0/43 |
| 2015 | 6/25 (24) | 5/16 (31.25) | 3/25 (12) | 0/16 | 0 | 0/16 |
| 2016 | 14/31 (45.2) | 11/33 (33.33) | 5/31(16.1) | 9/33 (27.27) | 0 | 0/33 |
| 2017 | 9/25 (36) | 5/22 (22.73) | 0 | 7/22 (31.82) | 0 | 1/22 (4.55) |
| 2018 | 6/25 (24) | 7/23 (30.43) | 5/25 (20) | 7/23 (30.43) | 2/25 (8.0) | 3/23 (13.04) |
| 2019 | 8/20 (40) | 7/28 (25) | 5/20 (25) | 4/28 (14.29) | 0 | 0/28 |
| 2020 | 7/17 (41.2) | 9/1 (11.11) | 3/17 (17.6) | 4/9 (44.44) | 0 | 0/9 |
